# Health-related quality of life of students from a private medical school in Brazil

**DOI:** 10.5116/ijme.563a.5dec

**Published:** 2015-11-08

**Authors:** Liliane Lins, Fernando M. Carvalho, Marta S. Menezes, Larissa Porto-Silva, Hannah Damasceno

**Affiliations:** 1 Department of Humanities, Bahiana School of Medicine and Public Health, Brazil; 2 Department of Social and Preventive Medicine, School of Medicine, Federal University of Bahia, Brazil

**Keywords:** Quality of life, medical students, medical schools, gov-ernment financing, training support

## Abstract

**Objectives:**

To assess health-related quality of life (HRQOL) and to describe factors associated with its variation among undergraduate medical students at a Brazilian private medical school.

**Methods:**

A cross-sectional study in a sample (n=180) of medical students at a private medical school in Salvador, Brazil, stratified by year of medical course. Data about age, sex, year of course, physical activity, sleepiness, headaches, participation in a student loan program supported by the Brazilian government (FIES) and living arrangements were collected using a self-administered form. HRQOL was assessed by using a Brazilian Portuguese version of the SF-36 form. The eight domains of SF-36 and the Physical Component (PCS) and Mental Component (MCS) Summaries scales were calculated.

**Results:**

The medical students showed poor HRQOL, mainly because of the mental component. Lower mean scores were found among those with FIES support, females, those suffering from sleepiness, headaches and lacking physical activity. No clear trend was observed in the variation of the SF-36 mean scores according to the year of medical school. However, students in the fifth year of the course had the highest HRQOL mean scores.

**Conclusions:**

Health-related quality of life of students at this private medical school was poor, mainly because of its mental component. Lower HRQOL was associated with FIES support, females, sleepiness, headaches and lack of regular physical activity. Higher scores were found among fifth year students.

## Introduction

The World Health Organization has defined quality of life as “individuals’ perception of their position in life in the context of the culture and value systems in which they live and their relation to their goals, expectations, standards and concerns”.^1^ Health-related quality of life (HRQOL) is a broad, multidimensional and polysemic concept that usually comprises subjective evaluations of positive and negative aspects of an individual’s physical and mental health. Health-related quality of life regards those aspects of self-perceived well-being that are related to or affected by the presence of disease, treatment and health policies.^2^

Because health-related quality of life is an inherently subjective concept, its correct measurement requires evaluation from the point of view of the affected individual.

The health-related quality of life of medical students can be affected by many occupational stressors.^3^ Having a perfectionist profile, being under great learning pressure, processing great amounts of new information, lacking time for social activities, and having contact with severe disease and with death can all contribute to the onset of depressive symptomatology among medical students.^4^ Depression, stress, and burnout were associated with low HRQOL among medical students.^5^  The undergraduate medical course seems to affect the health-related quality of life of the students, particularly their mental health component.^6^ A systematic review of 40 studies suggests a high prevalence of depression and anxiety among medical students, with levels of overall psychological distress consistently higher than in the general population and age-matched peers in the later years of training.^3^

Medical educators and health care personnel should pay attention to medical students’ mental health as most medical undergraduates report symptoms of mental illness, although of minor severity.^7^ Medical schools should monitor and evaluate the impact of the training period and curricular implementations on their students’ health-related quality of life.

In Brazil, 55.4% of the 240 medical schools are private; in 2014, they offered 58.2% of the 20,703 new places on medical courses.^8^   Since 2001, the Brazilian government has fostered an expensive loan program directed towards undergraduate students at private schools (FIES). Nowadays, approximately 20% of Brazilian medical students have a FIES loan. Financing varies from 50% to 100% of the monthly tuition fees depending on the proportion (20% to 60%) of the monthly family per capita income. The interest rate is 3.4% per year, with a period of grace of 18 months after the end of the financing period. The loan amortization period is three times the duration of the course plus twelve months.^9^ Therefore, the students who accumulate such a debt during the course face additional stress that could affect their health-related quality of life. 

The Short-Form 36 Questionnaire (SF-36) provides a generic, subjective measure of functional health and well-being from the individual's point of view. It has been extensively used for monitoring the health-related quality of life of specific populations with a wide range of diseases and health conditions.^10^ On October 9^th^ 2015, a PubMed search using the term “SF-36 survey” found 10,621 items.^11^ The SF-36 instrument has been validated in many countries and cultural contexts, among healthy and diseased people. Its sound methodological framework allows comparability among the several domains of the physical and mental aspects of the quality of life.

Several factors have been associated with health-related quality of life using SF-36 on medical students from Canada,^12^ Czech Republic,^13^ Iran,^14^^,^^15^ Serbia,^16^^,^^17^ Turkey,^18^ and Brazil^6^^,^^19^: These include sex, body mass index, year, academic performance, marital status, employment status, family income, living arrangements, number of reported diseases, smoking habit, alcohol intake, physical activity, and depressive symptoms. According to the SF-36 method, health-related quality of life comprises eight domains, considered multi-item scales: Physical Functioning (PF), Role limitations due to Physical problems (RP), Bodily Pain (BP), General Health perceptions (GH), Vitality (VT), Social Functioning (SF), Role limitations due to Emotional problems (RE), and Mental Health (MH). These eight scales can be aggregated into a Physical Component Summary (PCS) and a Mental Component Summary (MCS). According to the method recommended by the SF-36, comparability among the eight domains and the two components require score normalization because of their different variances. Unfortunately, most studies do not follow the strict methodological procedures recommended by the SF-36 developers,^20^ biasing their conclusions and/or impairing comparability.

This study aimed to assess health-related quality of life and to describe factors associated with its variation among undergraduate medical students at a Brazilian private medical school.

## Methods

### Study design and institution

In October/November 2013, a cross-sectional study was conducted among medical students at the Bahiana School of Medicine and Public Health (EBMSP), a private institution in Salvador, State of Bahia, Brazil. The EBMSP medical course offers 90-95 new places each semester. The full-time course takes six years to complete and awards a generalist physician diploma. 

### Sample size and sampling procedures

A total sample size of 180 students was arbitrarily and conveniently defined. A weighted stratified sampling procedure was applied according to each of the six-year classes.

### Data collection methods

Students were invited to fill in a self-administered questionnaire comprising: the SF-36 form; information about sociodemographic data, including participation in the study loan program; living arrangements; and aspects of health-related quality of life: sleepiness, defined by a score of 10 or more points in the Epworth Scale,^21^ headaches, and regular physical activity.  Data were collected during class intervals or after classes. Student participation was voluntary and the survey was anonymized. Whenever a student returned a questionnaire with a blank field, he/she was immediately invited to provide the information that was missing. 

The Ethical Review Board of the Bahiana School of Medicine and Public Health (Registration Number 233794/2013) approved this study.

This study used the validated Brazilian Portuguese 22 version of the SF-36 Health Survey version 1, as recommended by QualityMetric Incorporated.^20^ The questions in the SF-36 ask the students about health-related quality of life matters that have occurred in the last four-week period.

### Data processing and analysis

Data collected from the SF-36 form were used in the construction of eight scales: physical functioning, role limitations due to physical problems, bodily pain, general health perceptions, vitality, social functioning, role limitations due to emotional problems, and mental health. The eight multi-item scales were aggregated into Physical Component Summary and Mental Component Summary scores.

The scoring of the eight scales using the original 0 to 100 algorithms (raw score) and respective norm-based scores and the two summary measures was performed by QualityMetric Health OutcomesTM Scoring Software 4.0.^20^ Normalized scores enable comparisons to be made among the scales of the respective domain or component scale because of their comparable variance. This linear procedure transforms scores to a mean of 50 and standard deviation of 10 in the United States of America general population. This transformation achieves the same mean and standard deviation for all SF-36 scales and summary measures.^23^ Therefore, normalized scores enable unbiased identification and quantification of the SF-36 domains which are most affected by specific risk factors. Higher scores represent better health-related quality of life. This study was licensed by QualityMetric Health OutcomesTM with the number QM025904.

One-way analyses of variance, followed by Tukey’s tests when applicable, compared the means of SF-36 scales and its component scores according to course year. Spearman correlation coefficient measured the correlation between different years and SF-36 mean score scales (Table 2).

T-tests for independent samples were used to compare PCS and MCS mean scores stratified according to relevant covariates (Table 3). Data were analyzed using a set of programs provided by SPSS, version 21.0.24

## Results

One-hundred-eighty students filled in and returned questionnaires with complete and congruent data. There were no formal refusals.

Raw and normalized mean scores for the SF-36 eight domains, the Physical Component Score and the Mental Component Scores components are shown in Table 1.

**Table 1:** SF-36 raw and normalized scores (mean ± sd) of 180 students from a private medical school, Salvador, Brazil, 2013

Domain / summary component	Raw score	Normalized score
Physical Functioning (PF)	86.5 ± 16.3	51.5 ± 6.8
Role Physical (RP)	60.0 ± 39.8	44.9 ± 11.3
Bodily Pain (BP)	70.6 ± 21.2	50.2 ± 9.1
General Health (GH)	61.9 ± 15.4	46.2 ± 7.2
Vitality (VT)	50.0 ± 20.1	46.7 ± 9.5
Social Functioning (SF)	66.9 ± 24.5	42.7 ± 10.7
Role Emotional (RE)	56.5 ± 43.2	41.6 ± 13.6
Mental Health (MH)	67.9 ± 17.4	45.9 ± 9.9
Physical Component Summary (PCS)	50.4 ± 6.9	50.4 ± 6.9
Mental Component Summary (MCS)	42.0 ± 11.7	42.0 ± 11.7

In the fifth year, mean scores tended to reach a maximum in most scales and were particularly high for Vitality, Mental Health, and Mental Component Summary (Table 2). Spearman correlation coefficients between the course year and SF-36 domains and component summaries were very weak, none of them reaching a P-value < 0.10.

Physical Component Summary mean scores were significantly lower (P < 0.05 or less) among females, and among those who have FIES support, complaints of sleepiness and headaches. Mental Component Summary mean scores were significantly lower among those who have FIES support, complaints of headaches, and those who did not report regular physical activity (Table 3).

## Discussion

Among the 180 students, SF-36 normalized mean scores for Role Emotional (41.6 ± 13.6), Social Functioning (42.7 ± 10.7), and Mental Component Summary (42.0 ± 11.7) were far below 50.0, taken as a mean reference value from the United States of America general population (Table 1).

For the eight SF-36 domains scales, EBMSP students presented lower mean scores than people from two big studies with random samples in the city of Porto Alegre^25^ and from urban and rural areas of the five Brazilian regions.^26^ EBMSP students also presented lower Physical and Mental Component Summary mean scores than a sample of the general Brazilian population aged 18-24 years (Table 2).

Compared to medical students from a Brazilian public university (UNICAMP), 19 students from EBMSP presented lower mean scores on the scales that are more related to the physical component (PF, RP, BP, and GH), but scored higher in the scales more related to the mental component (VT, SF, RE, and MH). Compared to medical students from studies in Iran^14^^,^^15^ and the Philippines, ^5^EMBSP student scores varied widely with lower, higher, and equal mean scores according to the eight SF-36 domains.

Medical students from EBMSP showed poor health-related quality of life, mainly because of their mental component. An individual Mental Component Summary cutoff score of 42 is useful for detecting patients diagnosed with depressive disorder.^20^ The mean Mental Health Component Summary score of EBMSP students was 40.2 ± 11.7, suggesting that approximately half of these students were at risk of depression.

In our study, students scored very poorly in the Role Emotional (more problems with work or other daily activities as result of emotional problems) and Social Functioning

(more interference with normal social activities due to physical and emotional problems) domain scales. These two scales, together with the Mental Health scale, contribute most to the Mental Component Summary measure^27^ that was coherently low (42.0 ± 11.7).

The Physical Component Summary score of EBMSP students was similar to that found in the reference American population. However, both Physical Component Summary and Mental Component Summary were lower than those found among a reference Brazilian population from Porto Alegre.^25^

A reasonable explanation for these discrepant findings is that the comparison with the American reference population included all age groups, while the Brazilian population considered the specific age group 20-29 years.

**Table 2:** SF-36 normalized scores (mean ± sd) of students according to year of course in a private medical school, Salvador, Brazil, 2013

Domain/Component Summary	1^st^ Year (n=28)	2^nd^ Year (n=31)	3^rd^ Year (n=32)	4^th^ Year (n=32)	5^th^ Year (n=26)	6^th ^Year (n=31)
Physical Functioning (PF)	50.4 ± 5.9	53.0 ± 6.4	50.0 ± 8.1	49.9 ± 8.4	52.2 ± 5.7	53.6 ± 6.8
Role Physical (RP)	44.1 ± 10.0	46.2 ± 10.8	42.8 ± 11.8	44.3 ± 12.8	48.9 ± 10.7	43.9 ± 11.0
Bodily Pain (BP)	52.0 ± 9.2	49.6 ± 7.8	49.4 ± 9.3	48.3 ± 9.7	52.6 ± 9.6	49.8 ± 8.9
General Health (GH)	46.1 ± 7.0	45.7 ± 5.8	45.3 ± 7.1	45.1 ± 8.1	48.0 ± 7.4	47.1 ± 7.9
Vitality (VT)	47.0 ± 7.2	42.8 ± 8.5	45.1 ± 9.3	42.2 ± 9.1^¶^	52.1 ± 8.6^¶^	48.3 ± 11.7
Social Functioning (SF)	44.3 ± 11.0	41.1 ± 13.2	39.5 ± 11.5	40.9 ± 10.9	47.3 ± 10.3	42.8 ± 10.5
Role Emotional (RE)	44.8 ± 12.2	44.9 ± 7.8	39.2 ± 13.9	40.2 ± 14.6	46.4 ± 13.6	39.0 ± 13.6
Mental Health (MH)	48.0 ± 6.7	44.9 ± 7.8	44.8 ± 10.8	42.4 ± 12.1^‡^	51.8 ± 8.0^‡^	44.9 ± 10.2
Physical Component Summary (PCS)	49.2 ± 6.6	51.4 ± 7.2	49.3 ± 7.7	49.6 ± 6.7	51.3 ± 5.3	51.8 ± 7.4
Mental Component Summary (MCS)	45.1 ± 9.1	39.8 ± 11.3^*^	40.8 ± 12.1	38.7 ± 12.1^*^	48.5 ± 10.4^†^	40.4 ± 12.2

*, †: p < 0.05; ‡,‡: p < 0.005; ¶, ¶: p < 0.001.

We were unable to compare Physical and Mental Component Summary scores from our study with those reported by other studies, 14-17 because of the different ways of calculating these measures. Comparisons of scores of the eight domains between two different studies must be cautiously made.

Adequate comparisons among scores from different SF-36 domains, even among scales from a same study, require standardization that can be performed by using normalized-based scores. According to the SF-36 Users’Guide,^20^ the standardization of scoring is vital for score interpretation. Disregarding the recommended scoring method can invalidate meaningful comparisons of results across different studies and comparisons with scores for the general population. Most studies using SF-36 among medical students do not present normalized scores but “raw” (non-normalized) scores. Therefore, we could only compare our data with those from the literature using non-normalized scores, within each of the eight SF-36 scales.

Compared to students at a Brazilian public university,^19^ EBMSP students presented lower scores on the scales related to the physical component domains, but higher scores for all scales related to the mental domains of health-related quality of life.  However, compared to biomedical students from the Czech Republic,^13^ EBMSP had poorer health-related quality of life in all eight SF-36 domains. Consistent differences in health-related quality of life mean scores of these student populations were observed. Cultural differences among these studies, apart from the methodological ones already presented, could partially explain these findings.

We were unable to make useful comparisons between our study results and another study among Brazilian medical students^6^ that used SF-36, because the results were reported on the basis of the median and 25-75th percentiles.

In our study, we did not observe clear trends in the variation of the eight SF-36 domains nor in the component summaries over the six years of the medical course. However, the mental component of the health-related quality of life tended to decrease when comparing students from the first and in the sixth year of the course (Table 3).

Students in the fifth year of the medical course presented the highest mean scores of health-related quality of life. In the fifth year of the course, the theoretical content decreases markedly as internship training starts and students are not yet worried about the future competition that the medical residency selection process will bring about. Fifth year medical students at Brazilian public universities performed differently according to SF-36 scores. At the University of Uberlândia, Brazil, Year 5 medical students had better perceptions of their vitality in relation to HRQL compared with students in Years 2, 3, 4 and 66. However, medical students from the State University of Campinas, Brazil, scored significantly lower for the SF-36 domains mostly related to mental health-related quality of life: VT, SF, RE, and MH.^19^

Students in the final year of the medical course at a Brazilian public university presented lower median Mental Component Summary scores.^6^ During the final year of the medical school, Canadian students’ mean scores revealed a significant deterioration in vitality, role limitations due to physical problems, and role limitations due to emotional problems.^12^

Among EBMSP students, lower Physical Component Summary mean scores were significantly associated with females, those receiving FIES support, complaints of sleepiness, and complaints of headaches; lower Mental

Component Summary mean scores were significantly associated with FIES support, headaches and lack of regular physical activity (Table 3). As found in our study, female medical students from Iran^14^ presented lower PCS and MCs than males. Female medical students from a Brazilian public university presented lower median MCS than males.^6^

**Table 3:** SF-36 normalized component summaries scores (mean ± sd) according to covariates among 180 students from a private medical school, Salvador, Brazil, 2013

Covariates		n	Physical Component Summary	P	Mental Component Summary	P
Age, years						
	17-22	93	49.7 ± 7.1	0.154	42.1 ± 11.1	0.895
	23-33	87	51.2 ± 6.6		41.8 ± 12.3	
Sex						
	F	105	49.5 ± 7.2	0.031	40.7 ± 11.6	0.089
	M	75	51.7 ± 6.2		43.7 ± 11.5	
FIES						
	No	87	51.8 ± 6.6	0.010	43.8 ± 11.7	0.037
	Yes	93	49.2 ± 7.0		40.2 ± 11.4	
Sleepiness						
	No	87	51.8 ± 6.6	0.007	43.1 ± 11.9	0.194
	Yes	93	49.1 ± 7.0		40.9 ± 11.4	
Headache						
	No	84	52.3 ± 6.1	0.014	44.1 ± 10.7	0.001
	Yes	96	48.8 ± 7.2		40.1 ± 12.2	
Physical activity						
	No	73	49.6 ± 7.1	0.163	39.5 ± 12.1	0.018
	Yes	107	51.0 ± 9.7		43.6 ± 11.1	
Rented house						
	No	142	50.5 ± 7.1	0.691	42.5 ± 11.8	0.251
	Yes	38	50.1 ± 6.2		40.0 ± 10.8	
Live with family						
	No	48	50.8 ± 5.8	0.668	40.3 ± 11.4	0.264
	Yes	132	50.3 ± 7.3		42.5 ± 11.7	
Have a housemaid						
	No	74	49.8 ± 6.5	0.318	41.2 ± 11.6	0.444
	Yes	106	50.9 ± 7.2		42.5 ± 11.7	
Have a car						
	No	59	51.1 ± 7.4	0.398	40.0 ± 11.4	0.118
	Yes	121	50.1 ± 6.6		42.1 ± 1.79	

Sleepiness^28^ and headaches^29^ are frequent complaints among Brazilian medical students, as also observed in our study population. Among EBMSP students, headache complaints were associated with both physical and mental components of the health-related quality of life, while sleepiness complaints were strongly associated with the mental component alone.

Studies among Iranian medical students reported associations between lack of physical activity and lower PCS and MCS scores,^14^ and lower MCS scores.^15^  Low income is usually associated with poor health-related quality of life.^30^ According to Brazilian regulations, only students with a low per capita family income are eligible for a FIES loan. Therefore, a FIES loan can be taken as a proxy for family per capita income. A study among Brazilian medical students^6^ found no correlation between family income and Physical Component or Mental Component Summaries median scores. Studies in Iran^15^ and Serbia^17^ have reported associations between family income and HRQOL. However, their results are difficult to interpret because of methodological aspects.

### Limitations

It is necessary to comment on the strengths and weaknesses of this study. Cross-sectional design studies have intrinsic limitations and one of them is the difficulty in establishing the temporal relationship between dependent and independent variables. Furthermore, our student population was perhaps very homogenous according to family income, not allowing differences in subgroups to be revealed. Most of our student population could be classified as members of the middle to upper middle-class.

Apart from resource limitations, we had some difficulty calculating an adequate sample size for our study. When calculating sample sizes for studies involving SF-36, the following is recommended: a) to identify the dimension of primary interest upon which to base the sample size estimate and treat the others as secondary; or b) to identify the minimal clinically important difference (MCID).^31^ However, these two conditions did not fit into the mainly descriptive and exploratory purposes of our study. Furthermore, data about MCID for the SF-36 domains among medical students was not available.

The total sample size (n=180) in this study enabled us to detect statistically significant differences in mean scores, at a 95% confidence level, of effects varying ±1 % (when standard deviation = 6.8) and of effects varying ±2% (when standard deviation = 13.6).^32^

In spite of the possible limitations due to the small sample size increasing the probability of type II error, our study was capable of detecting significant differences between mean scores of groups of students classified according to several covariates. The use of corrections for multiple testing in an epidemiology study, such as Bonferroni’s procedure, have been strongly criticized.^33^

This study assessed physical activity and headache complaints in very simplistic ways.

Comparisons of our study results with those available in the literature were limited, because of their undue calculation and interpretation of SF-36 scores and summary measures.

## Conclusions

We may conclude that medical students at a private medical school showed poor health-related quality of life, mainly because of their scores in the mental component. Lower scores of health–related quality of life were associated with FIES student loan support, females, sleepiness, headaches and lack of physical activity. Literature about medical students’ health-related quality of life using SF-36 must be analyzed with caution, because of undue calculation and interpretation of its scores.

### Conflict of Interest

The authors declare that they have no conflict of interest.
